# The Global Prevalence of *Bacillus* spp. in Milk and Dairy Products: A Systematic Review and Meta-Analysis

**DOI:** 10.3390/foods14152599

**Published:** 2025-07-24

**Authors:** Tianmei Sun, Ran Wang, Yanan Sun, Xiaoxu Zhang, Chongtao Ge, Yixuan Li

**Affiliations:** 1 Key Laboratory of Precision Nutrition and Food Quality, Department of Nutrition and Health, China Agricultural University, Beijing 100083, China; usststm@163.com (T.S.); wangran@cau.edu.cn (R.W.); 15153515695@163.com (Y.S.); xiaoxuzhang0220@cau.edu.cn (X.Z.); 2 Aseptic Science and Technology, R&D AP, SIG Combibloc (Suzhou) Co., Ltd., Suzhou 215028, China

**Keywords:** *Bacillus* spp., random-effects model, milk, dairy products, microbial contamination, food spoilage

## Abstract

The spoilage of dairy products and foodborne diseases caused by *Bacillus* spp. are important public concerns. The objective of this study was to estimate the global prevalence of *Bacillus* spp. in a range of milk and dairy products by using a meta-analysis of literature data published between 2001 and 2023. A total of 3624 publications were collected from Web of Science and PubMed databases. Following the principles of systematic review, 417 sets of prevalence data were extracted from 142 eligible publications. Estimated by the random-effects model, the overall prevalence of *Bacillus* spp. in milk and dairy products was 11.8% (95% CI: 10.1–13.7%), with highly severe heterogeneity (94.8%). Subgroup analyses revealed substantial heterogeneity in *Bacillus* spp. prevalence according to geographical continents, sources of sampling, types of dairy products, microbial species, and detection methods. The prevalence of *Bacillus* spp. was highest in Asia (15.4%, 95% CI: 12.3–19.1%), lowest in Oceania (3.5%, 95% CI: 3.3–3.7%) and generally higher in developing versus developed countries. The prevalence of *Bacillus* spp. isolated from retail markets (16.1%, 95% CI: 13.0–19.7%) was higher than from farms (10.3%, 95% CI: 6.9–15.0%) or dairy plants (9.2%, 95% CI: 7.1–12.0%). This finding is likely attributable to its inherent characteristic of the resistant endospores and ubiquitous presence in the environment—*Bacillus* spp. can potentially cyclically contaminate farms, dairy products and human markets. Regarding the species distribution, *Bacillus cereus* presented a cosmopolitan distribution across all continents. The epidemic patterns of different *Bacillus* species vary depending on the sample sources. In addition, the detection method utilized also affected the reported prevalence of *Bacillus* spp. It is recommended to use molecular-based rapid detection methods to obtain a more accurate prevalence of *Bacillus* contamination. Therefore, a better understanding of variations in *Bacillus* spp. prevalence across different factors will enable competent authorities, industries, and other relevant stakeholders to tailor their interventions for effectively controlling *Bacillus* spp. in milk and dairy products.

## 1. Introduction

For centuries, dairy products such as milk, cheese, and yogurt have been recognized as essential components of the human diet, owing to their high nutrient content and moisture, and are consumed by people across all age groups worldwide [1,2]. Meanwhile, dairy products are also highly susceptible to microbial spoilage due to their favorable conditions for microbial growth, including the presence of nutritious proteins and fats [3]. The spoilage of dairy products caused by microbial contamination may result in substantial financial losses and damage to reputation [4]. In the dairy sector, spoilage causes losses of billions of dollars every year; in Europe, it accounts for 20% of the annual total production losses [5]. Furthermore, dairy products are a major source of bacteria-associated foodborne diseases, responsible for 4% of the global foodborne disease burden. This causes an annual economic burden of at least $4 billion in low- and middle-income countries [6,7]. Therefore, the need to increase knowledge on microbial contamination is fundamental to improving the quality of dairy products.

The contamination of milk and dairy products with endospore-forming bacteria is a daily challenge for the dairy industry. Members of the *Bacillus* genus with general characteristics of gram-positive, rod-shaped, aerobic and endospore-forming are among the most common bacteria encountered in the dairy environments [8,9]. Due to the abundant presence of these bacteria in the natural environment and their ability to form endospores, they can survive in various stress conditions, thereby maintaining endurance over a wide range of temperatures and pH values [10,11]. Moreover, highly resistant endospores of *Bacillus* species can protect them from food processing such as drying, pasteurization, etc. [12,13]. Germination and outgrowth of endospores of *Bacillus* species are not only associated with spoilage of milk but also responsible for foodborne diseases [14]. Among the endospore-forming *Bacillus* species, *Bacillus cereus* group is well known for its potential to cause two forms of food poisoning, namely the emetic and diarrheal syndromes [15]. Also, other *Bacillus* species, including *Bacillus licheniformis*, *Bacillus amyloliquefaciens*, and *Bacillus pumilus*, can produce toxic components such as surfactin, amylosin, and pumilacidin, contributing to food safety concerns [4,16,17]. Due to the potential hazard posed by *Bacillus* species in dairy products, there has been a substantial increase in related scientific publications. However, due to differences in regions, sampling plans, and detection methods, there is significant heterogeneity in the prevalence data [18]. It is thus critical to regularly review the published literature and obtain up-to-date insights to gain a comprehensive and systematic understanding of the prevalence of *Bacillus* spp. in milk and milk products worldwide.

Meta-analysis can use statistical approaches to combine the results from multiple individual studies on a specific research question. Additionally, meta-analysis can be used to identify the sources of heterogeneity or differences among findings of the primary studies [19]. In the past few years, meta-analysis has been increasingly applied to address a broad range of food safety issues such as disease incidence, prevalence and concentrations of microorganisms, effects of interventions, consumer practices, etc. [20,21,22,23,24]. The results from such independent meta-analyses can further provide valuable quantified estimates as input into microbial risk assessment models.

The present study was designed to use the meta-analysis tool to obtain an overview of the prevalence of *Bacillus* spp. in different types of milk and dairy products worldwide. It also aimed to delineate the distribution of *Bacillus* species, evaluate the variations in the prevalence of *Bacillus* in different geographical regions and determine the effect of microbial detection methods on the prevalence.

## 2. Materials and Methods

### 2.1. Search Strategy

The Preferred Reporting Items for Systematic reviews and Meta-Analyses (PRISMA) guidelines were used to perform this systematic review [25]. The research question guiding the data search was “estimate the prevalence of *Bacillus* spp. in milk and dairy products worldwide and evaluate the effects of different sampling factors on *Bacillus* spp. prevalence level”. A comprehensive search was performed for the prevalence of *Bacillus* spp. in two databases, i.e., Web of Science (WoS) and PubMed, using the same keywords. The following keywords were used as search queries in each database: “Bacillus” AND (“dairy products” OR “dairy” OR “milk products” OR “milk” OR “cream” OR “ice cream” OR “cheese*” OR “butter” OR “yogurt”) AND (“incidence” OR “prevalence” OR “prevalence rate*” OR “occurrence” OR “concentration” OR “contamination” OR “count” OR “survey” OR “sampling”). The search was limited to publication dates from 1 January 2000 to 31 December 2023. Publications with any of the selected keywords in the abstract, title, or main text were included.

### 2.2. Selection Criteria

During the screening stage of publications, in accordance with PRISMA guidelines, possible and suitable criteria regarding the objectives of the present study were considered in the screening and selection of obtained data [26]. After removing duplicate records, all the articles were checked according to a set of exclusion criteria. A study was excluded if (1) it was published as a conference abstract or was not a research paper (review); (2) it was not relevant, such as studies focusing on the detection method, predictive modeling, or hurdle technology; (3) it had overlapping information; (4) incomplete data on the prevalence and concentration of *Bacillus* spp. in dairy products were reported; (5) the sample size was lower than 20; (6) full text was not found. To minimize possible error, two researchers reviewed the publications separately.

### 2.3. Data Extraction

Data for *Bacillus* spp. prevalence on milk and dairy products were extracted from the studies identified through the systematic review of the literature independently by a single reviewer and validated by a second reviewer. The following data were extracted from each eligible study of records: author, publication year, survey year, dairy product category, sampling location (i.e., continent and country), source of sampling (i.e., farm, dairy plant, or retail market), detection method, sample size, number of positive samples, and identified *Bacillus* species. Subgroup analysis was conducted using the following potential moderator variables: types of milk and dairy products, geographic regions, sources of sampling, *Bacillus* species, and detection methods.

### 2.4. Meta-Analysis and Statistical Analyses

In this meta-analysis, the positive samples ( $p_{i}$) divided by the total sample size ( $n_{i}$) indicates the prevalence of *Bacillus* spp. in milk and dairy products ( I=pini). The pooled prevalence was calculated using random-effects models [with 95% confidence intervals (95% CI)]. Cochran’s Q test and the I-square index ( $I^{2}$) were used to detect heterogeneity between studies. The statistical significance for heterogeneity using Cochran’s Q test was defined as p<0.05, and the degree of heterogeneity using $I^{2}$ was defined as low, moderate, and high when $I^{2}$ values (as percentages) were around 25%, 50%, and 75% [27]. No publication bias is considered when the prevalence rate is obtained based on the ratio of positive sample size to total sample size [28,29]. Meta-analysis and forest plot generation in this study were conducted using R (version 4.4.3, http://www.R-project.org/ accessed on 1 January 2025), specifically the ‘meta’ package.

## 3. Results and Discussion

### 3.1. Systematic Review Process

**Figure 1:**
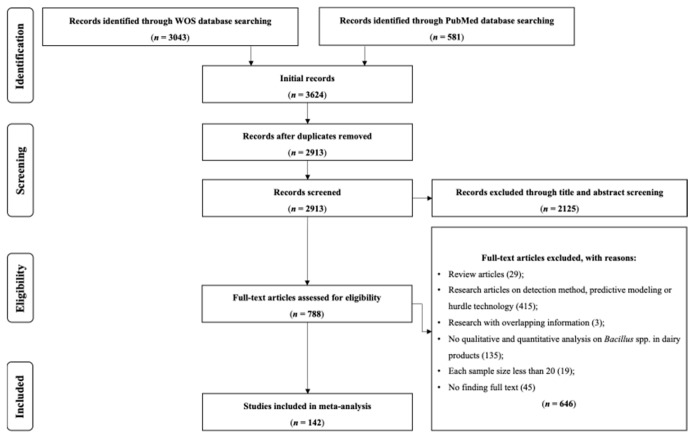
Flowchart of the literature search and data collection process.

Figure 1 summarizes the systematic review process conducted for this study. A total of 3624 publications were initially extracted from two selected databases. After deduplication, 2913 unique studies remained for relevance screening. Of the 2913 studies, 2125 were excluded during the title and abstract screening due to the lack of the mentioned inclusion criteria. A total of 788 studies were entered into the selection phase to be evaluated based on full text eligibility (inclusion and exclusion) criteria. Finally, 142 articles were deemed relevant with data successfully extracted. The included studies described *Bacillus* spp. prevalence in milk and dairy products in various countries all over the world.

### 3.2. Overall Prevalence of Bacillus spp.

Given the large number of included studies (142) and the wide range of dairy product type, Table 1 provides a summary of the meta-analysis. In this meta-analysis, a total of 54,772 samples were tested, including liquid milk, cheese, milk powder, ice cream, concentrated milk, other dairy products, and environmental samples. The overall pooled prevalence of *Bacillus* spp. in milk and dairy products was 11.8% (95% CI: 10.1–13.7%) based on all studies included in this global meta-analysis, with heterogeneity (as indicated by the inverse variance index) as high as 94.8%. Subsequently, subgroup analysis was conducted to identify the causes of heterogeneity.

### 3.3. Prevalence of Bacillus spp. Considering Geographic Regions

For the geographic region subgroup, the results showed that the rank order of pooled prevalence of *Bacillus* spp. in milk and dairy products was Asia > Africa > Europe > America > Oceania. The highest prevalence of *Bacillus* spp. was in Asia at 15.4% (95% CI: 12.3–19.1%) and the lowest prevalence was observed in Oceania at 3.5% (95% CI: 3.3–3.7%) (Table 2). The largest sample size was reported in studies across America. Conversely, Africa had the lowest number of included studies as well as the smallest sample size.

The prevalence of *Bacillus* spp. in milk and dairy products investigated in different countries varies substantially. A total of 42 countries from five continents were included in this study (see Supplementary Material Table S1). In Asia, Bangladesh showed the highest prevalence of *Bacillus* spp. in milk and dairy products (29.2%) and Korea showed the lowest (4.7%). In Africa, the prevalence of *Bacillus* spp. in dairy products was 13.9% (95% CI: 9.3–20.3%) which was the second-highest prevalence after Asia, although Africa had the fewest samples in this subgroup analysis. In Africa, the highest prevalence reported was in Ghana (39.1%), and the lowest was in Algeria (3.6%). In Europe, the pooled prevalence of *Bacillus* spp. was 11.4% (95% CI: 8.2–15.6%) which was the lowest after Africa. Among European countries, the highest prevalence of *Bacillus* spp. was reported in Germany and the lowest was in Britain. These study results, consistent with previous studies [11], show a higher prevalence of *Bacillus* spp. in food in developing countries (e.g., Ghana, Bangladesh, and Pakistan) compared to developed countries. Notably, exceptions were observed; Germany, despite being a developed country, reported a very high prevalence of *Bacillus* spp. contamination. Therefore, *Bacillus* spp. contamination also warrants attention in developed countries. The observed difference may be caused by variations in sample size, sampling method, environmental conditions, etc. [30]. In terms of environmental conditions, hygiene practices and climates (e.g., temperature, humidity) are the key factors influencing the prevalence of *Bacillus* spp. in both developing and developed countries. Poor hygienic conditions during milking, processing, and storage may significantly accelerate cross-contamination risks, thereby increasing the prevalence of *Bacillus* spp. [31]. Furthermore, warmer climates characteristic of tropical regions create favorable conditions for the growth and multiplication of microorganisms, potentially explaining the increased prevalence of *Bacillus* spp. observed in these geographical areas in this study [32,33,34]. Therefore, it is logical that the *Bacillus* prevalence in milk and dairy products varies between different geographical regions.

### 3.4. Prevalence of Bacillus spp. Considering the Source of Sampling and Type of Samples

In terms of sample sources, the prevalence of *Bacillus* spp. varied among samples from different sources. In this study, 92.64% (50,741 out of 54,772) of the total samples had definitive source information, among which 10,764 samples were isolated from farms, 24,581 samples were from dairy plants and 15,216 samples were from retail markets. As shown in Table 3, the pooled prevalence of *Bacillus* spp. isolated from retail markets (16.1%, 95% CI: 13.0–19.7%) was significantly higher than that from farms (10.3%, 95% CI: 6.9–15.0%) and dairy plants (9.2%, 95% CI: 7.1–12.0%). Due to their abundant presence in the natural environment, *Bacillus* spp. and their endospores have a high chance of circularly contaminating farms, dairy products, and human markets [35].

In the present study, we divided the types of included samples into seven categories: liquid milk, cheese, milk powder, ice cream, concentrated milk, other dairy products and environmental samples. The proportion of liquid milk samples was the largest (45.0%, 24,662/54,772), accounting for nearly half of the total included samples, followed by milk powder samples and cheese samples. Among the isolated samples from farms, the pooled prevalence of *Bacillus* spp. was relatively high in milk powder samples and environmental samples, which were 73.3% and 45.5% respectively. Though milk powder samples from farms showed the highest pooled prevalence result, it may need more investigations as only one study was included in this subgroup, which is insufficient in terms of representativeness [36]. The lack or the scarcity of representative research may generate a misconception about the real prevalence result [29]. By contrast, the significantly higher prevalence of *Bacillus* spp. in farm environmental samples is more conclusive. Its high prevalence may be due to the abundant presence of *Bacillus* endospores in the soil and consequent contamination of grass, feed, bedding material, air and cows [37]. This result is in accordance with findings of previous studies, which show that vegetables and cereals usually have a high prevalence of *Bacillus* species, indicating the impact from natural environmental factors such as soil contamination in agricultural products [11,38,39]. Thus, the environmental conditions of farms are one of the main sources of *Bacillus* spp. in dairy products. Strict farm management is necessary to decrease the contamination of *Bacillus* spp. at the farm level.

Among the isolated samples from dairy plants, the highest pooled prevalence of *Bacillus* spp. was found in cheese samples, at 24.2%. Likewise, *Bacillus* is also a major contaminant in retail ice cream and cheese samples. The pooled prevalence of *Bacillus* spp. contamination in retail ice cream and cheese samples was 44.2% and 17.6%, respectively. These findings further confirm that *Bacillus* species can grow and/or survive in foods with lower pH and at freezing temperatures. Cheese samples are a group of fermented milk-based food products that are produced in various flavors and forms around the world [40]. During the cheesemaking stage, the use of raw milk and the manufacturing process are the primary sources of microbial contamination [41,42]. A compilation of studies from 1973 to 2006, conducted in Switzerland, the United States, Sweden, Canada, France, Brazil, the United Kingdom, Spain, Malta, Scotland, England, and Finland, analyzed 84 cheese-associated outbreaks. The results showed that 69.0% of the outbreaks were linked to cheeses made from raw milk, while 7.2% involved cheeses without heat treatment [43]. In Brazil, 8% of the reported foodborne outbreaks related to artisanal cheeses made with raw milk were caused by *Bacillus cereus* contamination [44]. Furthermore, it is worth noting that the liquid milk samples from retail markets had a significantly higher prevalence of contamination than those sourced directly from farms or dairy plants. This elevated prevalence may also be related to the consumption of raw milk. Previous studies on milk consumption practices revealed that some people refrain from heating milk before consumption because they believe that heat treatment is detrimental to nutritional quality [45,46]. In several European countries, like France, Germany, and parts of the UK, raw milk may be available through numerous distribution channels, including direct sale to consumers at farms, sale through approved retailers, vending machines and the internet [47]. A study performed by Peng et al. indicated that raw milk posed higher contamination risks of aerobic *Bacillus*, with a prevalence of 54.02%, compared to pasteurized milk (14.41%) and sterilized milk (1.30%) [48]. Additionally, liquid milk samples in the retail markets often undergo temperature fluctuations during cold chain transportation and subsequent shelf-life storage, which may pose a risk for *Bacillus* spp. multiplication and recontamination [49]. Therefore, enhancing raw milk quality, ensuring adequate heat treatment, and maintaining cold chain transportation and storage are of great importance for achieving the quality and safety of dairy products.

Another reason for the high prevalence of *Bacillus* spp. in retail samples may be that dairy products are often sold in bulk or without aseptic packaging in some small-scale stores. Aseptic packaging, which involves sterilizing dairy products and sealing them in sterilized packages, has been growing in popularity due to its ability to enhance dairy safety and sustainability [50]. When at retail, unpackaged dairy products may be at a higher risk of exposure to microbial contamination during the handling process. Taken together, subgrouping based on the types of samples in addition to the source of samples can affect the pooled key measure levels.

### 3.5. Distribution of Bacillus Species

In this survey, a total of 35 species of the *Bacillus* genus were reported. The pooled prevalence calculation results for each *Bacillus* species in milk and dairy products are given in Table 4. Of these, *Bacillus sporothermodurans* (36.5%, 95% CI: 21.8–54.3%), followed by *Bacillus licheniformis* (26.3%, 95% CI: 16.0–40.1%) and *Bacillus cereus* (19.8%, 95% CI: 16.6–23.3%), are the most prevalent species contaminating milk and dairy products. Meanwhile, in terms of the number of studies and samples analyzed, *B. licheniformis* and *B. cereus* received considerable attention from researchers.

**Figure 2:**
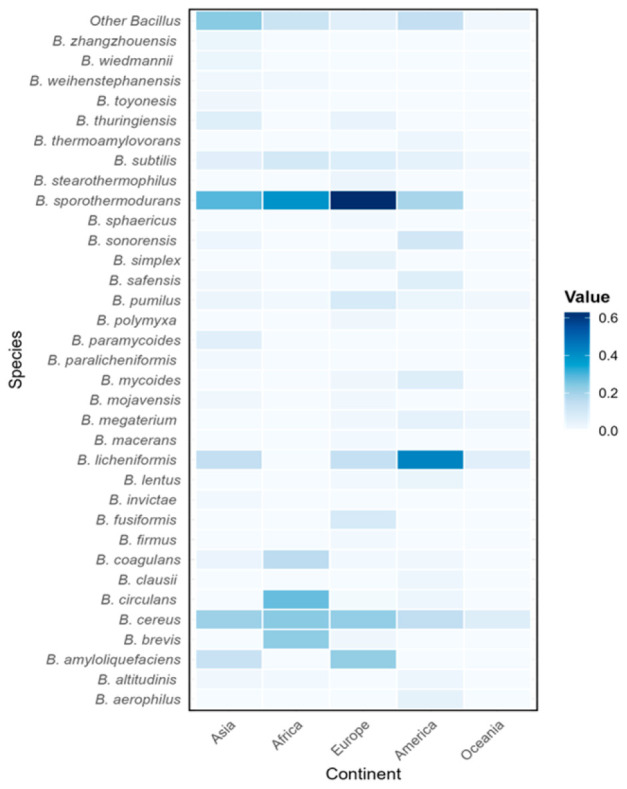
Worldwide distribution of *Bacillus* species by continents.

Figure 2 displays a worldwide distribution of *Bacillus* species by continents. *B. cereus*, *B. pumilus* and *B. subtilis* presented a ubiquitous profile, having been detected in milk and dairy products across all continents. Among these three species, the overall prevalence of *B. cereus* was the highest. This finding is supported by the fact that this species is commonly known for food pathogenicity within the *Bacillus* genus and has been reported in foodborne outbreaks worldwide. In the United States, *B. cereus* was linked to 1.74% of reported foodborne outbreaks from 1998 to 2008 [51]. In China, it accounted for 11.2% of outbreaks caused by pathogenic microorganisms between 2011 and 2016 [52]. In 2015, the European Food Safety Authority (EFSA) and the European Centre for Disease Prevention and Control (ECDC) reported that *B. cereus* ranked as the fourth most common cause of foodborne outbreaks in the European Union [53]. Food poisoning caused by *B. cereus* accounted for 17.8% of the total bacterial outbreaks in Finland, 11.5% in The Netherlands, 2.2% in Canada, 0.8% in Scotland, 0.7% in England and Wales, and 0.7% in Japan [54]. In addition, *B. subtilis* and *B. pumilus* have also been reported in connection with food poisoning due to their ability to produce toxic substances in food [55,56]. Nowadays, given the significant impact on public health of pathogenic *Bacillus* species in milk and dairy products, an increasing number of studies have been conducted to investigate their prevalence.

**Figure 3:**
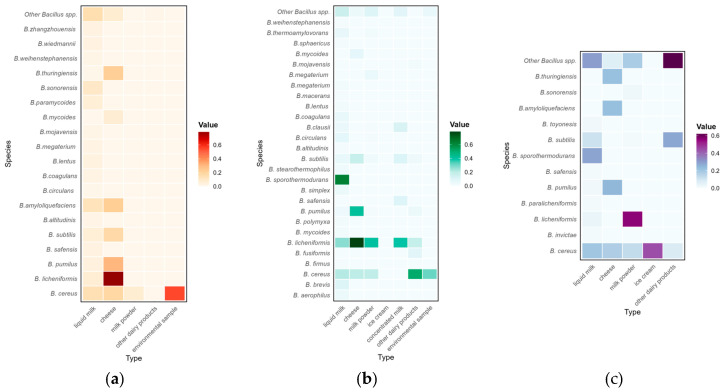
Distribution of *Bacillus* species across different types of milk and dairy products isolated from farms (**a**), dairy plants (**b**), and retail markets (**c**).

Figure 3 illustrates the distribution of *Bacillus* species in different types of dairy products from different sampling sources. Variations existed in the prevalence patterns of different *Bacillus* species depending on the sample sources. Overall, *B. cereus* and *B. licheniformis* were commonly detected in different types of samples across various sampling sources. In contrast, *B. sporothermodurans* was predominantly detected only in dairy plants and retail markets. According to the prevalence results, this study identified high-risk associations between *Bacillus* species and dairy products, including *B. cereus* in farm-sourced environments, *B. licheniformis* in farm-sourced cheese, *B. sporothermodurans* in dairy plant-sourced liquid milk products, *B. licheniformis* in dairy plant-sourced cheese, *B. cereus* in retail-sourced ice cream, and *B. licheniformis* in retail-sourced milk powder. On the other hand, these variations in this subgroup analysis may be related to the inherent characteristics of *Bacillus* endospores. Endospore-forming bacteria, especially *B. sporothermodurans* and *B. licheniformis*, can produce high thermoresistant endospores that can survive at an ultra-high temperature (UHT) [57]. The *D*_140 °C_-values of *B. sporothermodurans* endospores from UHT milk isolates vary between 3.4 and 7.9 s [58,59]. Furthermore, *B. cereus*, *B. sporothermodurans* and *B. licheniformis* also have been reported to have a strong ability to adhere to the surfaces of industrial equipment and form biofilms. The above characteristics help them survive and persist at all stages of farms, dairy plants and retailers, thus becoming frequently prevalent among different dairy products.

### 3.6. Prevalence of Bacillus spp. by Detection Methods

The detection methods of *Bacillus* spp. in the included studies were divided into three groups: culture–biochemical, culture–molecular, and culture–biochemical–molecular (see Table 5). The pooled prevalence of *Bacillus* spp. was calculated for each method. The results showed that the pooled prevalence of *Bacillus* spp. in milk and dairy products based on the detection method was 9.2% (95% CI: 7.4–11.3%) for the culture–biochemical method, 16.6% (95% CI: 13.1–20.8%) for the culture–molecular method, and 10.8% (95% CI: 6.6–17.0%) for the culture–biochemical–molecular method. This subgroup analysis based on detection methods indicated that this variable had a considerable effect on *Bacillus* prevalence in milk and dairy products.

As shown in Table 5, among the included studies, the culture-biochemical method was the most widely applied approach, having been used in the largest number of samples tested for *Bacillus* spp. This traditional biochemical method is known as the “gold standard” due to its sensitivity, low cost, and operational simplicity. However, this approach is also regarded as time-consuming and labor-intensive [60]. In addition, there is a probability that false negative results may occur due to viable but nonculturable cells (VBNC) [61]. Therefore, alternative molecular detection methods like polymerase chain reaction (PCR) methods are required to detect microorganisms more easily, quickly, and with high accuracy, reliability and sensitivity. The molecular methods are based on the amplification of specific DNA sequences and enable the detection of microorganisms even at low microbial concentrations [62]. Hence, the results of this subgroup analysis showed that the pooled prevalence of *Bacillus* spp. in milk and dairy products detected by molecular and biochemical-molecular methods was higher than that detected by the biochemical method alone. The use of molecular-based rapid detection methods for bacterial identification can provide more robust results.

## 4. Conclusions

In recent years, there has been a considerable rise in the demand for milk and dairy products. Compared with other foods, milk and dairy products are particularly susceptible to microbial contamination throughout the farm-to-table chain and are subject to rapid spoilage and foodborne diseases. In the present systematic review, the global prevalence of *Bacillus* spp. in milk and dairy products based on defined subgroups was meta-analyzed. The results revealed that the prevalence of *Bacillus* spp. in developing countries is higher than that in most developed countries. The prevalence and species distribution of *Bacillus* spp. also varied among different types of milk and dairy products from different sources. Notably, *B. cereus*, as a pathogenic species within the *Bacillus* genus, was found to be widespread across all continents and in diverse dairy products globally, posing a greater potential risk to consumers. These findings underscore the imperative of implementing stringent, enforceable, and monitorable hygiene standards (covering facilities, equipment, processes, personnel, and the environment) to prevent the spread of contamination by *Bacillus* spp. and their endospores. However, due to the lack of detailed sampling information (e.g., the time when dairy products leave the processing plant, transport time, storage time in retail, etc.) limiting this study, it is impossible to further predict dynamic contamination patterns of *Bacillus* species. Implementing rigorous microbial sampling and monitoring frameworks coupled with more accurate detection methods, such as PCR and other molecular techniques, can yield more precise prevalence data. In the future, the integration of such data can better assess the ultimate exposure and risk of *Bacillus* species in dairy products and assist government authorities, industry, and other relevant stakeholders in enhancing food safety and preventing new outbreaks.

## Figures and Tables

**Table 1:** Summarized information of the meta-analysis of *Bacillus* spp. prevalence in dairy products.

Total Included Study	Total Inputs	Total Sample Size	Pooled Prevalence (95% CI) ^a^	*τ*^2^ ^b^	*I*^2^ ^c^
142	417	54,772	11.8% (10.1–13.7%)	2.9600	94.80%

^a^ 95% CI: 95% confidence interval; ^b^ *τ*^2^: between-study variance; ^c^ *I*^2^: inverse variance index.

**Table 2:** Prevalence and sample size of *Bacillus* spp. by continent.

Continents	Total Inputs	Total Sample Size	Pooled Prevalence (95% CI) ^a^	*τ*^2^ ^b^	*I*^2^ ^c^
Asia	134	13,953	15.4% (12.3–19.1%)	2.1207	92.30%
Africa	31	2252	13.9% (9.3–20.3%)	1.4542	86.20%
Europe	110	12,841	11.4% (8.2–15.6%)	3.4255	94.90%
America	123	19,096	10.2% (7.5–13.7%)	3.1815	95.60%
Oceania	13	2877	3.5% (3.3–3.7%)	5.4501	96.00%
Unknown	6	3753	3.1% (0.5–16.0%)	4.8592	98.80%

^a^ 95% CI: 95% confidence interval; ^b^ *τ*^2^: between-study variance; ^c^ *I*^2^: inverse variance index.

**Table 3:** Prevalence and sample size of *Bacillus* spp. by different types of samples from different sources.

Sampling Site	Sample Type	Total Inputs	Total Sample Size	Pooled Prevalence(95% CI) ^a^	*τ*^2^ ^b^	*I*^2^ ^c^
Farm	Liquid milk	77	9606	7.6% (5.1–11.2%)	3.3014	94.40%
	Cheese	2	105	20.8% (7.3–46.7%)	0.6157	90.30%
	Milk powder	1	30	73.3% (55.0–86.1%)	-	-
	Other dairy products	1	27	0.0%	-	-
	Environmental sample	12	906	45.5% (20.1–72.8%)	4.0493	94.00%
**Total**	**/**	**93**	**10,674**	**10.3% (6.9–15.0%)**	**4.1614**	**94.60%**
Dairy plant	Liquid milk	76	9492	8.6% (6.0–12.2%)	2.4552	94.70%
	Cheese	15	865	24.2% (10.7–46.0%)	3.5303	94.60%
	Milk powder	49	13,356	5.9% (3.3–10.5%)	4.3748	97.10%
	Ice cream	1	24	0.0%	-	-
	Concentrated milk	9	209	14.6% (8.2–24.4%)	0.5367	60.50%
	Other dairy products	11	584	19.7% (9.1–37.6%)	2.0681	90.30%
	Environmental sample	6	321	13.4% (3.9–37.0%)	2.3417	93.20%
**Total**	**/**	**167**	**24,851**	**9.2% (7.1–12.0%)**	**3.2256**	**95.40%**
Retail	Liquid milk	60	4171	16.4% (12.0–22.1%)	1.8553	88.90%
	Cheese	35	2853	17.6% (12.7–23.7%)	1.1223	85.50%
	Milk powder	26	4472	12.9% (7.0–22.4%)	2.7663	96.70%
	Ice cream	6	1012	44.2% (28.3–61.4%)	0.6456	88.70%
	Other dairy products	15	2708	10.9% (4.7–23.2%)	3.0115	93.20%
**Total**	**/**	**142**	**15,216**	**16.1% (13.0–19.7%)**	**2.0203**	**93.50%**

^a^ 95% CI: 95% confidence interval; ^b^ *τ*^2^: between-study variance; ^c^ *I*^2^: inverse variance index.

**Table 4:** Prevalence and sample size of *Bacillus* spp. by microbial species.

Species	Total Inputs	Total Sample Size	Pooled Prevalence (95% CI) ^a^	*τ*^2^ ^b^	*I*^2^ ^c^
*Bacillus cereus*	210	21,837	19.8% (16.6–23.3%)	2.2394	93.50%
*Bacillus licheniformis*	32	6027	26.3% (16.0–40.1%)	3.1015	94.40%
*Bacillus pumilus*	25	4165	3.1% (1.6–5.9%)	2.2891	88.10%
*Bacillus safensis*	5	311	3.6% (1.4–9.2%)	0.6948	64.60%
*Bacillus subtilis*	31	5184	5.3% (3.4–8.2%)	1.4281	91.40%
*Bacillus altitudinis*	3	232	1.7% (0.7–4.5%)	0.0000	0.00%
*Bacillus mojavensis*	2	135	1.5% (0.4–5.7%)	0.0000	0.00%
*Bacillus wiedmannii*	1	66	3.0% (0.8–11.3%)	-	-
*Bacillus weihenstephanensis*	1	66	1.5% (0.2–1.0%)	-	-
*Bacillus zhangzhouensis*	1	66	3.0% (0.7–11.3%)	-	-
*Bacillus paramycoides*	1	66	6.1% (2.3–15.1%)	-	-
*Bacillus sonorensis*	3	906	5.7% (2.4–12.9%)	0.4974	90.30%
*Bacillus circulans*	6	1212	3.4% (0.7–14.6%)	3.3753	95.10%
*Bacillus coagulans*	7	1102	3.1% (1.0–9.0%)	1.7724	88.90%
*Bacilus weithenstephanensis*	1	83	1.2% (0.2–8.1%)	-	-
*Bacillus amyloliquefaciens*	2	58	17.2% (9.5–29.2%)	0.0000	0.00%
*Bacillus thuringiensis*	3	321	4.52% (0.65–25.5%)	2.5422	88.40%
*Bacillus mycoides*	2	169	3.6% (1.4–8.8%)	0.1241	60.10%
*Bacillus megaterium*	4	759	2.9% (1.8–4.7%)	0.0449	32.10%
*Bacillus lentus*	3	195	2.1% (0.8–5.3%)	0.0000	0.00%
*Bacillus sporothermodurans*	4	483	36.5% (21.8–54.3%)	0.5024	95.00%
*Bacillus clausii*	7	1570	2.4% (1.0–5.8%)	1.0418	70.70%
*Bacillus brevis*	3	240	10.2% (2.2–37.1%)	1.8577	92.20%
*Bacillus paralicheniformis*	1	114	0.9% (0.1–6.0%)	-	-
*Bacillus toyonesis*	1	114	1.8% (0.4–6.7%)	-	-
*Bacillus invictae*	1	114	0.9% (0.1–6.0%)	-	-
*Bacillus thermoamylovorans*	5	1523	2.5% (0.5–11.3%)	2.6330	88.00%
*Bacillus simplex*	1	22	4.6% (0.6–26.2%)	-	-
*Bacillus fusiformis*	1	69	8.7% (4.0–18.0%)	-	-
*Bacillus sphaericus*	1	111	0.9% (0.1–6.1%)	-	-
*Bacillus macerans*	1	111	0.9% (0.1–6.1%)	-	-
*Bacillus firmus*	1	111	0.9% (0.1–6.1%)	-	-
*Bacillus polymyxa*	1	111	1.8% (0.5–7.0%)	-	-
*Bacillus stearothermophilus*	1	111	2.7% (0.9–8.0%)	-	-
*Bacillus aerophilus*	1	22	4.6% (0.6–26.2%)	-	-
*Other Bacillus*	44	6986	11.9% (7.8–17.6%)	2.1392	95.30%

^a^ 95% CI: 95% confidence interval; ^b^ *τ*^2^: between-study variance; ^c^ *I*^2^: inverse variance index.

**Table 5:** Prevalence and sample size of *Bacillus* spp. by detection method.

Detection Method	Total Inputs	Total Sample Size	Pooled Prevalence (95% CI) ^a^	*τ*^2^ ^b^	*I*^2^ ^c^
Culture–biochemical	222	34,572	9.2% (7.4–11.3%)	2.8320	95.50%
Culture–molecular	165	17,428	16.6% (13.1–20.8%)	3.0342	93.70%
Culture–biochemical–molecular	30	2772	10.8% (6.6–17.0%)	1.9548	91.00%

^a^ 95% CI: 95% confidence interval; ^b^ *τ*^2^: between-study variance; ^c^ *I*^2^: inverse variance index.

## Data Availability

No new data were created or analyzed in this study. Data sharing is not applicable to this article.

## Supplementary Materials

The following supporting information can be downloaded at: https://www.mdpi.com/article/10.3390/foods14152599/s1, Table S1: Prevalence and sample size of *Bacillus* spp. based on countries.
