# Supplementary material for: The Global Prevalence of Bacillus spp. in Milk and Dairy Products: A Systematic Review and Meta-Analysis
Source: Foods. 2025 Jul 24;14(15):2599. doi: 10.3390/foods14152599 (PMC12346675; doi:10.3390/foods14152599)
Supplement: Supplementary file 1 [file foods-14-02599-s001.zip › Table S1.pdf]

Table S1. Prevalence and sample size of *Bacillus* spp. based on countries.

Country	Total inputs	Total sample size	Pooled prevalence (95% CI) [a]	τ^{2} [b]	I^{2} [c]
China	55	9,920	11.4% (7.6-16.8%)	2.6719	95.70%
Brazil	32	3,139	12.0% (6.6-20.9%)	3.2809	91.70%
Colombia	4	300	42.3% (12.2-79.4%)	2.7762	97.10%
Canada	1	250	40.8% (34.9-47.0%)	-	-
Italy	16	1,730	21.3% (13.8-31.2%)	0.9772	92.50%
Multiple countries	5	3,710	1.9% (0.3-11.9%)	4.5924	99.10%
Greece	5	644	16.4% (7.9-30.9%)	0.7248	89.80%
Algeria	8	664	3.6% (1.3-9.5%)	1.6096	84.10%
Mexico	2	75	0% (0-1%)	0.0000	0.00%
Ethiopia	2	182	17.6% (4.2-51.3%)	1.2026	94.90%
India	4	170	26.7% (19.8-34.9%)	0.0192	42.00%
Bangladesh	6	175	29.2% (4.9-76.7%)	6.0838	77.80%
Australia	9	2,681	1.7% (0.4-8.1%)	5.3120	96.10%
Turkey	29	3,271	2.3% (1.4-3.9%)	1.6214	83.20%
Malawi	2	114	14.0% (8.8-21.7%)	0.0000	39.90%
Ghana	5	210	39.1% (22.6-58.4)	0.6616	86.70%
Bolivia	1	43	7.0% (2.3-19.5%)	-	-
Egypt	55	2,638	18.8% (14.3-24.5%)	1.3682	84.20%
New Zealand	4	196	15.5% (3.3-49.6%)	2.6343	93.70%
America	73	1,4196	7.9% (5.4-11.5%)	2.8579	96.40%
Ireland	31	1,975	28.7% (17.7-43.0%)	2.8813	92.90%
Czech Republic	11	1,896	12.7% (4.9-29.1%)	2.5583	94.80%
Spain	4	171	16.8% (5.2-42.3%)	1.2673	64.30%
Iran	8	521	15.7% (7.1-31.2%)	1.4025	89.90%
Tanzania	1	128	6.3% (3.2-12.0%)	-	-
Nigera	1	23	21.7% (9.4-42.8%)	-	-
Saudi Arabia	2	262	28.9% (17.1-44.6%)	0.2028	91.60%
South Africa	1	74	39.2% (28.8-50.7%)	-	-
Finland	1	39	5.1% (1.3-18.3%)	-	-
Bauchi State	1	214	19.6% (14.8-25.5%)	-	-
Tunisia	1	84	4.76% (1.8-12.0%)	-	-
Korea	3	147	4.7% (0.2-50.0%)	5.2668	69.10%
Burkina-Faso	1	43	9.3% (3.5-22.3%)	-	-
Botswana	8	516	19.4% (13.0-27.9%)	0.3540	80.10%
England	2	1,295	0.3% (0.1-0.8%)	0.0000	0.00%
Unknown	1	43	25.6% (14.8-40.5%)	-	-
Denmark	1	75	9.3% (4.5-18.3%)	-	-
Poland	9	936	20.6% (12.5-32.2%)	0.7489	89.50%

Table S1 *Cont.*

Malaysia	1	120	20.8% (14.5-29.0%)	-	-
Chile	5	215	45.6% (36.5-55.0%)	0.0817	54.20%
Argentina	1	50	40.0% (27.5-54.0%)	-	-
Uruguay	4	828	8.0% (2.3-23.9%)	1.6253	97.70%
Germany	1	809	62.8% (59.4-66.1%)	-	-

[a] 95% CI: 95% confidence interval; [b] τ^2: between-study variance; [c] I^2: inverse variance index.
